# Pharmacological Potential of 3-Benzazepines in NMDAR-Linked Pathophysiological Processes

**DOI:** 10.3390/biomedicines11051367

**Published:** 2023-05-05

**Authors:** Nadine Ritter, Paul Disse, Bernhard Wünsch, Guiscard Seebohm, Nathalie Strutz-Seebohm

**Affiliations:** 1 Institute for Genetics of Heart Diseases (IfGH), Department of Cardiovascular Medicine, University Hospital Münster, D-48149 Münster, Germany; paul.disse@uni-muenster.de (P.D.); guiscard.seebohm@ukmuenster.de (G.S.); nathalie.strutz-seebohm@ukmuenster.de (N.S.-S.); 2 Chembion, University of Münster, D-48149 Münster, Germany; wuensch@uni-muenster.de; 3 Institute of Pharmaceutical and Medicinal Chemistry, University of Münster, Corrensstr. 48, D-48149 Münster, Germany

**Keywords:** ionotropic glutamate receptors, neurodegeneration, Alzheimer’s disease, GluN2B, 3-benzazepines

## Abstract

The number of *N*-Methyl-D-aspartate receptor (NMDAR) linked neurodegenerative diseases such as Alzheimer’s disease and dementia is constantly increasing. This is partly due to demographic change and presents new challenges to societies. To date, there are no effective treatment options. Current medications are nonselective and can lead to unwanted side effects in patients. A promising therapeutic approach is the targeted inhibition of NMDARs in the brain. NMDARs containing different subunits and splice variants display different physiological properties and play a crucial role in learning and memory, as well as in inflammatory or injury processes. They become overactivated during the course of the disease, leading to nerve cell death. Until now, there has been a lack of understanding of the general functions of the receptor and the mechanism of inhibition, which need to be understood in order to develop inhibitors. Ideal compounds should be highly targeted and even splice-variant-selective. However, a potent and splice-variant-selective NMDAR-targeting drug has yet to be developed. Recently developed 3-benzazepines are promising inhibitors for further drug development. The NMDAR splice variants GluN1-1b-4b carry a 21-amino-acid-long, flexible exon 5. Exon 5 lowers the NMDAR’s sensitivity to allosteric modulators by probably acting as an NMDAR modulator itself. The role of exon 5 in NMDAR modulation is still poorly understood. In this review, we summarize the structure and pharmacological relevance of tetrahydro-3-benzazepines.

## 1. NMDARs and Neurodegeneration

At least 50 million people worldwide suffer from Alzheimer’s disease or dementia. In 2019, 121,500 deaths were attributable to Alzheimer’s disease, making this disease the sixth leading cause of death in the United States [1]. Alzheimer’s disease is characterized by progressive cognitive decline, which is triggered by the degeneration of neurons and their synapses and the resulting brain atrophy. This corresponding cognitive decline is detectable in humans as well as in animal models. Increasing concentrations of amyloid β (Aβ) and tau proteins are suspected to be responsible for this neurotoxicity [2]. In the functioning brain, tau proteins are responsible for supporting cell skeletons by forming microtubules. Aβ is a large membrane protein that, in a healthy state, plays an important role in the growth and repair of neurons. Phosphorylation of tau proteins can result in aggregation and misfolding, which ultimately leads to neurodegeneration [3]. When Aβs are misfolded, they stack and form the plaques typical for Alzheimer’s disease, which then lead to neurodegeneration [4]. Currently, there are no effective treatments or prevention options for Alzheimer’s disease. Understanding the mechanisms underlying synaptic degeneration is key to developing treatments.

**Figure 1:**
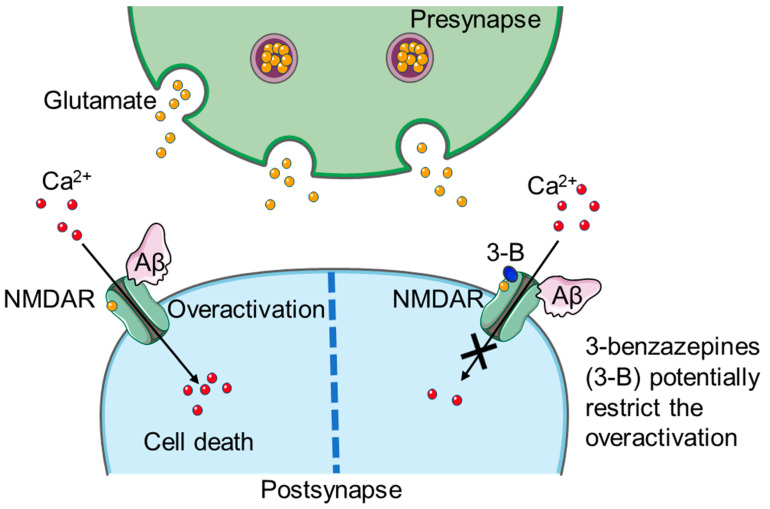
Mechanism of NMDAR overactivation by Aβ (light pink) at the postsynapse (light blue) and the potential decrease in overactivation by 3-benzazepines (dark blue). During stimulus conduction, glutamate (yellow) is released as a neurotransmitter from vesicles (purple) into the synaptic cleft via exocytosis. Glutamate can then activate postsynaptic glutamatergic receptors. Soluble Aβ (light pink) can interact with NMDARs (turquoise), which in turn leads to excitotoxicity due to increased calcium (red) influx, followed by apoptosis induction and neurodegeneration. Figure created partly with Servier Medical Art (Creative Commons Attribution 3.0 Unported License).

It was found that oligomeric Aβ plays a role in inducing excitotoxicity at the terminal of the presynapse by mediating calcium influx into the synapse and by forming membrane pores, which mediates further calcium influx [2,4]. Misfolded tau proteins in presynaptic terminals can lead to the degradation of synaptic vesicle proteins such as synaptotagmin, synaptogyrin 3, and synaptophysin, resulting in the exhaustion of synaptic vesicle pools [2]. Moreover, a higher number of malfunctioning mitochondria is observed in the presynapse linked with Alzheimer’s disease, thus promoting the production of reactive oxygen species (ROS). Postsynaptic ROS production is also linked to mitochondrial malfunction. α-Secretases cleave amyloid precursor protein (APP) and the soluble APPα form, which is part of the nonamyloidogenic pathway. It is suggested that soluble Aβ is able to bind various receptors. This induces the activation of the tyrosine-protein kinase Fyn, which further enhances inositol triphosphate (IP_3_) and eukaryotic elongation factor 2 (eEF2), thus leading to the phosphorylation of synaptic tau. This Fyn activation is also linked to cyclic increases and decreases in NMDARs [2]. However, soluble Aβ oligomers have been shown to interact with *N*-Methyl-D-aspartate receptors (NMDARs), which belong to the group of ionotropic glutamate receptors (Figure 1) [5]. Misfolding and phosphorylation of tau proteins can also be caused by the binding of Aβ to NMDARs, which in turn leads to excitotoxicity due to calcium influx, followed by a reduction in cAMP response element-binding protein (CREB) and disinhibition of GSK3β [2].

NMDARs are essential for excitatory neurotransmission [6]. At pH 7.3, protons inhibit NMDARs by about 50%. During ischemia and seizures, pH decreases by 0.2–1.0 pH units, causing glutamate release to activate the NMDAR. Neuronal death can result from this overactivation and represents a threat to the central nervous system (CNS). Alkalization, on the other hand, reduces calcium influx through the NMDAR, thereby acting neuroprotective [7]. The basis of learning and memory formation is formed by upregulated NMDAR expression, which in turn mediates increased calcium influx, leading to more activity, which is called Long-Term Potentiation (LTP) [8]. As a second messenger, calcium reinforces synaptic LTP by triggering postsynaptic signaling pathways. When calcium binds to calcium calmodulin-dependent protein kinase II (CaMKII), it initiates phosphorylation events that often cause LTP [9].

Alterations in NMDARs can result in a loss of synapse function. It was found that oligomeric Aβ interacts with the postsynaptic TMEM97 (σ_2_ receptor), which can then cause calcium dysregulation causing neurodegeneration. By activating IP_3_ and ryanodine receptors, dendritic spines can also release intracellular calcium from the smooth endoplasmic reticulum. Eventually, the amyloidogenic pathway is initiated when postsynaptic APP is cleaved via γ-secretases into Aβ, which leads to toxic soluble Aβ oligomers and ultimately Aβ plaques [2]. Understanding the basic functions and mechanisms of NMDARs is key to developing drugs that can restrict overactivation and eventually lead to the treatment of Alzheimer’s disease.

However, NMDARs can also be involved in other diseases. According to Milnerwood et al. (2010) [10], neuronal death may also be caused by NMDAR-mediated excitotoxicity in some chronic neurodegenerative diseases, such as Huntington’s disease. The primary cause of neuronal death after ischemia or injury in stroke and TBI is apparently NMDAR-dependent excitotoxicity, and NMDAR blockers protect neurons from ischemic cell death in vitro and in vivo [11,12,13]. Additionally, altered glutamate signaling may build a pathophysiologic basis for schizophrenia [14]. Further, there is a link between the dysfunction of NMDARs and depression. Overstimulation of NMDARs may be the cause of major depression [15]. Additionally, glutamatergic transmission plays a crucial role in addiction [16].

## 2. NMDARs as a Class of Ionotropic Glutamate Receptors

Ionotropic glutamate receptor functions are important for several processes in the brain, spinal cord, retina, and peripheral nervous system. Thus, their dysregulation plays a key role in different neurological diseases [17]. These glutamate receptors can be found in all types of neuronal cells [18]. Ionotropic glutamate receptors (iGluRs) are homo- or heterotetrameric membrane proteins with four subunits, which altogether form the ion pore and mediate the ion transport. In recent studies, it has been reported that iGluRs form extensive molecular complexes (>400 kDa) [17,19]. There are three distinct classes of iGluRs: α-amino-3-hydroxy-5-methyl-4-isoxazolepropionic acid receptors (AMPARs), NMDA, and Kainate receptors (KainateRs). The three receptor types are expressed postsynaptically in the CNS and are associated with LTP, synaptic plasticity, and stimulus conductance. The initial nomenclature referred to activating agonists, but molecular cloning revealed that each class has multiple subunits encoded genetically. The NMDAR is named after its substrate, *N*-methyl-D-aspartate [18]. NMDARs, in contrast to other glutamate receptors, are voltage-dependently blocked by external magnesium, have a high calcium permeability, and require the binding of two co-agonists to activate the receptor [17,20].

The neurotransmitters bind to the excitatory synapses and open the cation channels, which depolarizes the neuron. Postsynaptic neurons are depolarized by a glutamate-driven iGluR inward current [20,21]. Electrical events caused by postsynaptic stimulation are called excitatory postsynaptic currents (EPSCs) [22]. An EPSC is primarily induced by the activation of AMPARs, followed by the activation of NMDARs. AMPARs mediate a fast (approximately one ms) and NMDARs a slow synaptic current (ten to hundreds of ms) [20,21,23]. Following the release of glutamate into synaptic clefts, glutamate concentrations increase rapidly for a short period, then decay with a time constant of approximately 1.2 ms due to the diffusion and active removal of glutamate by glutamate transporters [20]. Glutamate binding to AMPARs (and/or KainateRs) and NMDARs results in conformational changes that enable the ion channel pores to open (gating). EPSCs mediated by NMDARs continue to pass a current for tens to hundreds of milliseconds after the synaptic glutamate is removed. The reason for this is partly due to receptor binding affinity, but also because receptor activation requires pregating and repeated transitions between glutamate-bound open and closed conformational states, where glutamate eventually unbinds and EPSCs end [20,24,25]. At rest, magnesium blocks NMDARs’ pores under physiological conditions. AMPAR activation can release this block upon depolarization. Since NMDARs are voltage-dependent and ligand-gated, they can be used as detectors for both presynaptic glutamate release and postsynaptic depolarization. Further, this results in calcium influx into the dendritic spine and subsequently leads to short- or long-term synaptic changes [17,20].

## 3. The Different Domains of the Heterotetrameric NMDAR

**Figure 2:**
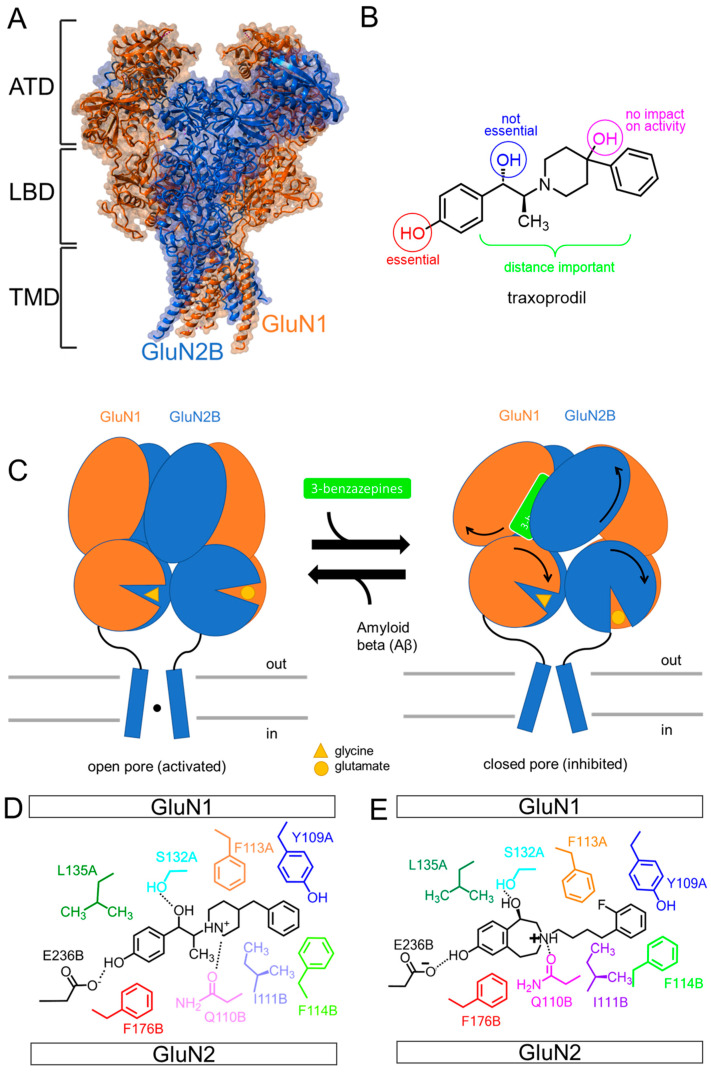
The NMDAR structure and the respective inhibitory mechanism are essential for drug development. NMDAR structure with GluN1 (orange) and GluN2 (blue) subunits, formed as a dimer of dimers containing ATD, LBD, and TMD (PDB ID: 6CNA, edited with YASARA structure 20) (**A**). Relevant and nonrelevant structural modifications for highly selective NMDAR modulators shown via traxoprodil as ifenprodil derivative. The length of the alkyl spacer has a major impact on NMDAR inhibition (green), while the benzylic OH^−^ group was not required for the activity (blue). A hydroxy group on one of the benzenes is a crucial component of an active compound (red). The OH moiety in 4-position of the piperidine ring had no impact on NMDAR activity (pink) (**B**). Rolling motion of the ATDs and LBDs facilitate the opening and closing of the NMDAR-associated ion channel (**C**). GluN2B-containing NMDARs are activated by glycine and glutamate (yellow) binding and inhibited by the rolling of LBDs, which is influenced by ATD conformational changes. The rolling motions modify the tension on the TMDs and allow the receptor to open or close. When the LBDs roll down, the tension on the linkers between the LBD and TMD decreases, and the NMDAR eventually closes. Soluble Aβ overactivates the NMDAR, and 3-benzazepine compounds inhibit the NMDAR. The ifenprodil binding pocket is located at the GluN1 (orange) and GluN2B (blue) interface. Further, 3-bezanzepines (green) can inhibit via this NMDAR modulation site. (**D**,**E**) Shown is the ifenprodil binding pocket with ifenprodil (**D**) and (*R*)-OF-NB1 (**E**) bound. (*R*)-OF-NB1 was obtained by introducing the F-atom in the 4-phenylbutyl side chain of WMS-14-10. Aromatic, H-bond, and hydrophobic interactions between the 3-benzazepine and the ifenprodil binding pocket lead to inhibition.

NMDARs are heterotetrameric receptors and formed as a dimer of dimers. They contain different GluN subunits and consist of two glycine-binding GluN1 and two glutamate-binding GluN2 subunits [20,26,27,28]. NMDARs comprise an amino-terminal domain (ATD), an extracellular ligand-binding domain (LBD), a transmembrane domain (TMD) with a pore loop region (Figure 2A), and a carboxy-terminal domain (CTD) [18,29]. Several loops of the LBD point towards the extracellular membrane, partially covering the extracellular end of the TMD. LBDs are formed by local GluN1/GluN2B heterodimers. As a result of the cross-over between ATD and LBD, ATD subunits from one heterodimer connected with LBD subunits from another heterodimer form a bulky extracellular domain. TMD segments are further spatially united by M4 helices interacting nearly exclusively with TM segments from a neighboring subunit [28].

### 3.1. Amino-Terminal Domain (ATD)

In total, the ATD of NMDARs consists of about 400 amino acids. Two subdomains in the ATD, R1 and R2, form a tertiary structure resembling an open shell (clamshell-like) [30]. In contrast to AMPARs and KainateRs, NMDARs have a larger ATD subunit interface with the LBD, which modifies the receptor’s gating. The R2 lobes of the ATD interact with the LBDs, and the R1 lobes of the ATD bind to compounds such as Ro25-6981 at the ATD subunit interface. The S1-S1 interface between heterodimers (GluN1 and GluN2B) displays a major interface within the LBD layer. Another interface is built within heterodimers. NMDARs are allosterically modulated at the S1-S1 interface of intramers [28,31,32,33]. The ATD R2 lobes of the GluN2B subunits are located near the symmetry axis, while those of the GluN1 subunits are located on the periphery of the assembled receptor. GluN1-ATD heterodimers are shaped like an inverted ‘V’, with the open end located towards the GluN1 LBD, and loops and residues of GluN1 R2 interacting with its GluN1 LBD. GluN2B R2 interacts with the interdimer LBD interface [28].

### 3.2. Ligand-Binding Domain (LBD)

In the LBD layer, GluN1 and GluN2B subunits form two major interfaces between dimers and the S1-S1 interface. NMDARs exhibit allosteric modulation at their intradimer S1-S1 interface. Two nearly identical interdimer interfaces are observed between heterodimeric LBDs, including GluN1 interacting with GluN2B loop 1 and GluN2B helix–helix interactions [28,31,33]. Both GluN1 and GluN2B subunits interact with residues on their S1 and S2 lobes, providing a direct route to modulate the LBD clamshell closure, which is translated into LBD layer rearrangement [28]. GluN1/GluN2B homodimers form contacts with their cognate LBD subunits, with the R2 lobes of each GluN1 subunit interconnecting extensively with the S1 lobes of the cognate GluN1 LBD and the R2 lobes of each GluN2B subunit extending into the LBD dimer–dimer interface to make contacts with both the cognate GluN2B LBD and its nearest GluN1 LBD [28]. Thus, the ATDs are positioned to influence conformational transitions at both interdimer and intradimer interfaces [28,31,33].

### 3.3. Transmembrane Domain (TMD)

The TMD consists of four segments (M1-M4) and an intracellular CTD. A ‘collar,’ which surrounds the extracellular NMDAR M3 helices, is formed by NMDAR pre-M1 vesicles. The M1 subunit traverses the membrane and interacts with both the M3 helix of the pore-lining subunit and the neighboring M4 helix. At the turn between M2 and the selective filter’s starting point, asparagine residues facilitate the voltage-dependent magnesium block. These residues project their side chains into the cytoplasmic space [28,34]. Extracellularly, a pyramid-shaped M3 segment exhibits mechanical constriction for the permeation of ions. The M4 segment interacts primarily with the M1 and M3 helices of a nearby subunit, thereby extending its turns. Furthermore, the M4 segment resides on the periphery of the TMD, interacting mainly with the M1 and M3 helices of a neighboring subunit and extending for several turns extracellularly [28].

## 4. Properties of NMDAR Subunits

Unlike AMPARs and KainateRs, NMDARs display domain-switches and are composed of different GluN subunits. Currently, 18 types of ionotropic glutamate receptor subunits are known. Seven of these eighteen subunits have been identified for the NMDAR: GluN1, GluN2A-D, and GluN3A-B (Table 1). Each of these subunits consists of approximately 900 to 1400 individual amino acids; thus, a complete NMDAR comprises over 4000 amino acids. Seven genes encode for the various NMDAR subunits: a single *grin1* gene for GluN1, four *grin2* genes for GluN2A-D, and two *grin3* genes for GluN3A-B. *Grin1* has eight splice variants: 1a-4a and 1b-4b. Moreover, three exons can be spliced: exons 5, 21, and 22. Exon 5 is encoded by 21 amino acids (^191^SKKRNYENLDQLSYDNKRGPK^211^) and is located in the GluN1-1b-4b ATD. NMDARs, including exon 5, show a decreased agonist sensitivity [20]. Exon 5 shields the NMDAR from proton inhibition [35]. Moreover, exon 5 creates an interdomain contact between the LBD of the GluN1 and GluN2 subunits and stabilizes the dimer interface [36]. Exon 5 was observed to lower the proton sensitivity of NMDARs, probably by stabilizing both the ATD-LBD and the LBD-LBD interfaces. Within exon 5 of GluN1-1b, two amino acids were identified to interact with nearby GluN2B and GluN1 amino acids (GluN1-1b K190 interacts with GluN2B Y507 and GluN1-1b K211 interacts with GluN1 D786). NMDARs containing exon 5 were observed to display a lower allosteric modulator sensitivity (zinc, ifenprodil, and spermine) [20]. Further, exon 5 increases the rate of deactivation of the NMDARs. Taken together, it is likely that exon 5 acts as a modulator of the NMDAR [35,37]. Additionally, exon 5 was found to be involved in colonic inflammation processes, i.e., colitis. NMDARs carrying exon 5 were upregulated 14, 21, and 28 days after colitis induction in rat spinal cords. Processes such as inflammation result in a local pH drop, and exon 5 decreases pH sensitivity. Expressing NMDARs containing exon 5 may increase the NMDAR activity during inflammation [38].

The ATD domain is linked to the LBD by a GluN1 α4-β7 loop and an α5-helix, which further terminates at exon 5 in alternatively spliced GluN1b variants [39,40]. Exon 5 is encoded by an N1 cassette in the *grin1* gene. The C-terminus of the GluN2B α4 helix and the α4-β7 loop are prone to NMDAR modulation by polyamines [41]. While linking peptides connecting the ATDs to the LBDs plays a significant part in the transduction of conformational changes between the two layers, direct interactions that harness the expected large-scale motions of the ATDs also play a central role in conveying changes to the TMD (Figure 2C) [28,32,42].

The N1 cassette, which codes for exon 5, affects protein kinase C sensitivity [43,44]. In all four GluN1-b splice variants, the N1 cassette is located in the ATD R2 subdomain. Alternative splicing of exons 21 and 22, encoded by the C1 and C2 cassettes, alters NMDAR expression and regulation [44]. The C0 cassette is not altered during splicing. Both GluN1-1a and GluN1-1b have C0, C1, and C2 cassettes in their C-terminal domains [44]. The C1 cassette is absent in GluN1-2a and GluN1-2b [45]. The first stop codon of GluN1 is deleted when the C2 cassette is removed by splicing exon 22, resulting in an alternative sequence that is constrained by a second stop codon after 66 base pairs (22 amino acids). The GluN1-4a and GluN1-4b splice variants, both of which have the C2 cassette, also lack the C1 cassette. The C2 and C1 cassettes are both present in GluN1-3a and GluN1-3b [46].

Spermine and spermidine reduce desensitization in NMDARs. In whole-cell patch-clamp recordings at physiological magnesium concentrations, spermine increased the desensitization of NMDARs containing GluN1-1a/GluN2B. However, this was not observed in cells transfected with GluN1-1b/GluN2B. Polyamines and exon 5 have similar effects on the proton sensitivity of NMDARs, which led to the identification of structural parallels between polyamines and the exon 5 surface loop. Exon 5 and spermine induced faster deactivating responses, possibly due to decreased entry, accelerated desensitization recovery, and reduced affinity. Spermine accelerated the recovery from desensitization in GluN1-1a/GluN2B responses and decreased the entry into desensitization with GluN1-1a/GluN2B responses but not GluN1-1b/GluN2B responses. Due to decreased entry and accelerated recovery from desensitization, it was discovered that exon 5 and spermine produced faster deactivating responses [37]. Agonist affinity determines rapid deactivation and is largely dependent on unbinding [24]. The observed decrease in agonist affinity for exon 5 or spermine might contribute to faster deactivation as well [47]. Exon 5 and spermine both shortened the fast decay components of (*S*)-glutamate responses. Slower deactivation processes could lead to continuous activation of NMDA and a constitutive calcium influx in neurons, which might promote apoptosis. Consistent with this hypothesis, the death of retinal ganglion cells following optic nerve crush is linked with increased expression of GluN1-1a [37].

The GluN2 expression is affected by spatial and developmental factors. Since the different GluN2 subunits impart different properties to the GluN receptor, various diseases are attributed to the NMDAR due to dysfunctions of the GluN2 subunits [48]. In rodents, GluN2B is highly expressed during embryonic and postnatal development as well as in adult brains. The GluN2A subunit is expressed strongly from the second postnatal week of development onwards, and together with GluN2B it is the most prevalent NMDAR subunit in adult forebrains [49]. GluN2B subunits are the most widespread NMDAR subunits in the adult forebrain, which is why this subunit is a key target for drug treatment of Alzheimer’s disease [20,26,27,28]. Specifically, GluN2B subunits are associated with neurodegenerative diseases such as Parkinson’s disease, Alzheimer’s disease, and Huntington’s disease because they are mostly located extrasynaptically and promote high calcium conductances, which can induce apoptosis. Diseases including schizophrenia, anxiety disorders, and depression are more associated with GluN2A subunits [50,51,52]. GluN2A subunits are dominant during brain maturation, especially at postsynaptic sites, where they form diheteromeric (GluN1/GluN2A) and triheteromeric (GluN1/GluN2A/GluN2B) NMDARs. While GluN2D subunits are likewise strongly expressed during the development of the brain, their expression steadily declines in comparison with the GluN2B subunits [53]. GluN2C is extensively expressed in but not restricted to the cerebellum. Only early on in development, the brain stem, the cerebellum, and the diencephalon display a high GluN2D expression. The GluN2C subunit, in turn, is linked to white matter injury and consequently involved in multiple sclerosis [54].

**Table 1:** The seven different NMDAR subunits and their respective properties and roles in neurodegeneration (adapted from Paoletti et al. 2013 [55]).

Subunit		Function	Role in Neurodegeneration
GluN1		One subunit/gene has eight distinct splice variants, which influence the gating and pharmacological properties of NMDARs. Glycine binding.	Via alternative splicing, some splice variants have embedded structures acting like ligands, which decrease proton sensitivity. Therefore, the NMDAR function can be maintained during pH drops, occurring during, i.e., seizures.
GluN2	A	Alteration of NMDAR function by glycine dependency. Expresses strongly from the second postnatal week of development and is the most prevalent NMDAR subunit in the adult forebrain [49]. Can form both diheteromeric (GluN1/GluN2A) and triheteromeric (GluN1/GluN2A/GluN2B) NMDARs. The deactivation rate is the fastest, and magnesium sensitivity as well as calcium permeability is highest in NMDARs consisting of GluN2A.	GluN2A subunits are dispersed from synaptic sites in anti-NMDAR encephalitis. Schizophrenia is associated with reduced NMDAR activity and/or presence in inhibitory GABAergic neurons, resulting in an imbalance of activity in the neuronal network. Further, there was a loss of GluN2A-containing receptors described. In Parkinson’s disease, synaptic GluN2A expression is increased.
	B	GluN2B-containing NMDARs are enriched at extrasynaptic sites, and the expression decreases during the brain’s development. GluN2B NMDARs show a higher sensitivity to voltage-dependent magnesium block, higher calcium permeability, and higher single-channel conductance compared with GluN1/GluN2C and GluN1/GluN2D NMDARs. Glutamate binding. GluN2B subunits are one of the most prevalent types of NMDAR subunits in the adult forebrain.	During cerebral ischemia and traumatic brain injury, overaction of extrasynaptic NMDARs leads to neuronal death. Major alterations by overexpression and phosphorylation of GluN2B-containing receptors lead to (chronic) pain. The synaptic relocation of GluN2B-containing receptors is associated with Parkinson’s disease. The overactivation of GluN2B-containing NMDARs mediates Aβ-induced alterations in synaptic plasticity and synapse loss, which results in excitotoxicity in Alzheimer’s disease. Cognitive decline is associated with reduced expression of NMDARs, particularly GluN2B-containing receptors. Depressive symptoms are reduced rapidly (within hours) and sustainably (within days) by NMDAR inhibitors.
	C	GluN2C is extensively, however not exclusively, expressed in the cerebellum. In general, this subunit’s expression is lower than GluN2A and 2B. GluN2C has a higher GluN2 agonist potency than GluN2A and 2B.	In white matter injuries, the activity of GluN2C-NMDARs results in oligodendrocyte damage and demyelination. Thus, GluN2C plays a role in multiple sclerosis.
	D	Functional NMDARs containing GluN2D at lower abundance are present at an older age, particularly in the basal ganglia. GluN2D subunits are expressed at lower levels. Of all GluN2 subunits, GluN2D displays the highest GluN2 agonist potency.	GluN2D might mimic GluN2B-induced overactivation following excitotoxicity in stroke [56].
GluN3	A, B	Coexpression of GluN1 and GluN3 subunits results in a glycine-gated excitatory NMDAR. NMDARs containing GluN3 display reduced magnesium sensitivity, calcium permeability, and decreased agonist-induced current responses.	GluN3A subunits might also have an impact in multiple sclerosis through oligodendrocyte damage and demyelination.

## 5. The Prototypic GluN2B Inhibitor Ifenprodil

Ifenprodil (2-(4-benzylpiperidin-1-yl)-1-(4-hydroxyphenyl)-propan-1-ol), an active ingredient contained in vasodilator drugs (Cerocral, Dilvax, Vadilex), has been known since 1971 [57]. It was discovered that the alkyl spacer had a major impact on NMDAR inhibition, with IC_50_ of 178.4 nM and binding affinities of 10 nM for GluN1-1a/GluN2B-containing receptors [58,59]. However, the benzylic OH^−^ group was not required for the activity. The alkyl spacer between position 4 of the piperidine ring and the terminal benzene ring on the right-hand side of the molecule can be extended or shortened to increase the NMDAR potency of the compound (longer spacers lead to less active compounds) (Figure 2B). It is essential to connect two benzene rings with a spacer of a specific length. A hydroxy group on one of the benzenes was another crucial component of the active compounds. The presence of another hydroxyl group at position 4 of the piperidine ring strengthened the separation of these activities [57]. The potency was slightly decreased by modifying the spacer between the two terminal benzene rings [60]. In traxoprodil (GluN1-1a/GluN2B IC_50_ 11 nM [61]), a compound derived from ifenprodil, which has an OH moiety in 4-position of the piperidine ring, it was shown that this OH moiety had no impact on NMDAR activity (Figure 2B) [57,60,62]. Traxoprodil was developed for the treatment of major depression. However, due to cardiovascular side effects the clinical trial was discontinued [62].

Ifenprodil stabilizes the binding of glutamate on NMDARs with a low open probability while inhibiting the binding of glycine allosterically. Additionally, ifenprodil blocks the spermine binding site sensor and stabilizes the desensitized state of the NMDAR [7]. In spite of this, ifenprodil has a low target selectivity and can also bind to both α-adrenergic and some serotonergic receptors [57]. Therefore, several other phenylethanolamine derivatives were investigated (e.g., Ro 8-4304) that showed higher selectivity for NMDARs containing a GluN2B subunit. It is likely that the inhibition is mediated by alkalizing [7].

**Figure 3:**
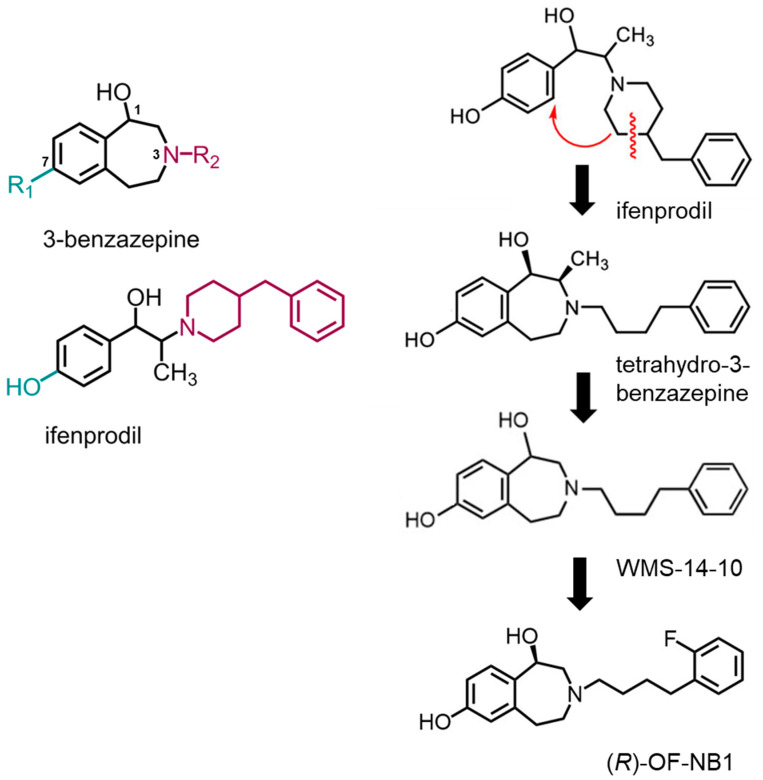
Ifenprodil as a precursor for the synthesis of 3-benzazepine derivatives. All generated 3-benzazepine derivatives were side-chain-modified (N-R2, dark purple) (**left**). The deconstruction of ifenprodil as a leading compound to the tetrahydro-3-benzazepine to WMS-14-10 and eventually (*R*)-OF-NB1 (**right**). Ifenprodil and the tetrahydro-3-benzazepine are constitutional isomers. The methyl moiety and the phenolic and benzylic OH moieties were removed to retrieve WMS-14-10. Adapted from Ritter et al. 2021 [65].

Therefore, other ifenprodil derivatives were synthesized, and their inhibitory effects and modulatory effects on NMDARs were investigated. The ifenprodil-derived 3-benzazepine WMS-1410 (with an IC_50_ of 18.4 nM and binding affinities of 84 nM for GluN1-1a/GluN2B [63,64]) was shown to be a potent antagonist of NMDA receptors with a GluN2B subunit [65]. A structurally modified 3-benzazepine antagonist targeting NMDARs was investigated using WMS-1410 as a lead compound. A tetrahydro-3-benzazepine, representing a constitutional isomer of ifenprodil, was synthesized and side-chain-modified (Figure 3). The resulting tetrahydro-3-benzazepine has a lower binding affinity but a higher inhibitory activity on NMDARs containing GluN1/GluN2B subunits in electrophysiological experiments than ifenprodil. As a result of further removing the phenolic and benzylic OH^−^ moieties, WMS-1410 was obtained, which also exhibited GluN1/GluN2B inhibition and affinity [65,66]. In order to inhibit NMDARs, one of the two OH^−^ groups in 3-benzazepine antagonists must be present [67]. Modification in the tetramethylene spacer or its benzene ring and testing of these compounds revealed various potent NMDAR inhibitors, i.e., (*R*)-OF-NB1, (*S*)-OF-NB1, and PF-NB1. Furthermore, the inhibitory activity of these identified compounds was confirmed on human induced pluripotent stem cell (hiPSC) derived glutamatergic neurons as a physiological test system [59]. (*R*)-OF-NB1, a WMS-1410 derivative, was established as a selective NMDAR antagonist with significant NMDAR splice variant preference [68]. (*R*)-OF-NB1 and PF-NB1 were developed successfully as GluN2B-selective and powerful inhibitors (IC_50_ for GluN1-1a/GluN2B of 97 nM and 60.9 nM [59,68]), and their application as positron emission tomography (PET) tracers was confirmed [69,70,71,72,73]. Because of its high blood–brain barrier permeability, ^18^F-OF-NB1 builds up in areas of the brain where GluN2B is abundant. When compared with ^18^F-PF-NB1, ^18^F-OF-NB1 showed greater cerebellum accumulation in ex vivo biodistribution experiments [70,71,72,74,75]. Regarding this, ^18^F-OF-NB1 is a promising GluN2B radioligand that is appropriate for PET imaging studies in ALS patients and those with other neurodegenerative diseases [72].

Ifenprodil binds to a distinct binding pocket located at the interface of GluN1 and GluN2B in the ATD (Figure 2D). Several ligand-binding pocket interactions, including aromatic, hydrogen bond, and hydrophobic interactions, have been demonstrated to play a significant role in this binding site [67]. The inhibitory mechanism was determined by identifying five Interaction Zones (IZs) within the ifenprodil binding site. A strong inhibitory impact of NMDARs is dependent on interactions in IZ1, 2, 3, and 5. The amino acid E236 in the IZ1 forms a hydrogen bond with the phenolic group of the ligand in the GluN2B subunit. The hydrophobic and hydrogen bond interactions between GluN1 L135 and GluN1 S132, the benzylic hydroxyl group, and the 3-benzazepine form IZ2. The GluN2B subunit’s aromatic interactions with amino acids F114 and F176 are the major basis for IZ3 and IZ5. Hydrophobic interactions of GluN2B I111 and GluN1 F113 and the compound, as well as hydrogen bond interactions between compound and residue Q110 in the GluN2B subunit, form IZ4. Moreover, ifenprodil exhibits an aromatic interaction with the GluN2B subunit of the NMDAR at position F176 [67]. Several GluN2B selective ligands can be assigned to different groups. The interaction between the A-ring and the binding pocket is consistent across a large group of structurally diverse ligands. Additionally, there is a structurally distinct linker between the two aryl moieties, allowing for different ligand-binding pocket interactions. B-rings exhibit the most diverse interactions between ligands and binding sites. According to current pharmacophore models, the activity of the highly potent compound depends on π/π interactions with the aromatic amino acid GluN2B F176 [76]. Fluorine weakens cation-π interactions. Since fluorine withdraws electrons, the electron density is reduced at the aromatic ring [77]. The fluorine in (*R*)-OF-NB1 probably acts through-space by cation-π interaction deactivation at the aromatic ring of GluN1 F113 (Figure 2E) [68].

## 6. Downstream Allosteric Modulation of NMDARs

Allosteric interactions enable structural distance communication and represent a basic key biological concept. Allosteric interactions describe distinct structural regions in a protein whose conformational spaces are interlinked [78]. In the NMDARs described here, binding sites in the receptors undergo reversible conformational changes, which is known as allosteric transition. Allosteric modulation plays an important role in cell signaling and intercellular communication and is particularly important for the regulation of ligand-gated ion channels’ functions, such as NMDARs [6,79,80,81]. Allosteric transitions occur in ligand-gated ion channels where chemical energy (binding neurotransmitters) is successfully transferred mechanically (opening the ion channel pores via TMD). Allosteric modulation sites tightly regulate these channels and receptors and regulate their activity and activation with so-called allosteric modulators [6,79,81]. Molecular selectable and highly druggable allosteric modulation sites are crucial in pharmacology and therapeutics [6,80,82]. As previously described, the NMDAR comprises four extracellular domains, displaying interfaces and having large bilobate (clamshell-like) extracellular domains, the LBDs [6,27,28,83,84,85]. Tight conformational ATD-LBD coupling is likely to result in NMDAR ATDs’ allosteric signaling (opening probability, kinetics) [6,31]. Furthermore, the distinct binding sites of the NMDAR ATD provide several possibilities for small molecule regulations and interactions [6,86,87]. Allosteric modulators probably work by altering the conformational equilibrium between the active and inactive states of the ATD dimers, thereby enhancing or attenuating receptor activity [5,41,83,84,88]. Activation of the NMDAR requires interdomain mobility [6].

According to published cryo-EM data on the GluN1/GluN2B receptor, the two LBD dimers undergo an extensive rotation as the receptor changes from an inactive to an active form [84]. Mutagenesis and cross-linking indicate that the rolling motion between two pairs of LBD heterodimers is an essential part of the receptor selection mechanism [6]. LBD rolling at the interdimer interface acts as a gate switch controlling channel opening and as a pivotal allosteric transition that links the upper ATD region with the receptor’s gating core. NMDAR activation can be modified by reorienting the two LBD dimers in the GluN1/GluN2 region of the NMDAR, which is translated by a physical mechanism. The interdimer rolling of the LBD causes the ATD to enter an active state. Positive allosteric modulators such as spermine, which bind to the GluN1-GluN2B ATD interface, stabilize the compact conformation of the receptor, promote rolling, and thereby enhance receptor activity. In contrast, negative allosteric modulators such as zinc and ifenprodil inhibit receptor activity by stabilizing the extended form of the ATD by pushing the lower lobes further apart and thus preventing the rolling motion. The LBD-TMD-connecting linkers facilitate pore opening by rolling motions [6].

## 7. Conclusions

Altogether, NMDARs are of high scientific and clinical interest and represent prime pharmacological targets. Ideal compounds display highly targeted and even splice variant specificity. The drug memantine is already approved as an NMDAR blocker for the treatment of moderate to severe Alzheimer’s dementia. However, memantine blocks all NMDARs, resulting in side effects. Selective modulation of NMDARs with particular subunits such as, e.g., the GluN2B subunit, could reduce the side effects. The latest advances in highly selective NMDAR drug research offer highly promising approaches as starting points for drug development. However, these compounds must still be further optimized. The understanding of the unique regulation of NMDARs by naturally embedded structures such as exon 5, as well as binding and the downstream allosteric network, starting from ATD to LBD and the eventual closing and opening of the pore in the TMD, is of importance for the development of appropriate drugs. Via this approach, positive therapeutic effects can be maximized, and negative side effects might be reduced, improving drug therapy for patients. Eventually, these optimized lead compounds need to be tested in further established and optimized disease models, such as hiPSC-derived neuron 3D neuronal networks and SK-N-SH cells [89]. In terms of safety pharmacology, these modulators must be investigated on common off-targets. (*R*)-OF-NB1, an optimized 3-benzazepine derived from ifenprodil, can be used as a positive example in drug development, leading to a powerful imaging tool in ALS and potentially other neurodegeneration diseases [68,70,72,73].

## Data Availability

No new data were created or analyzed in this study. Data sharing is not applicable to this article.
